# Modelling Co-Infection with Malaria and Lymphatic Filariasis

**DOI:** 10.1371/journal.pcbi.1003096

**Published:** 2013-06-13

**Authors:** Hannah C. Slater, Manoj Gambhir, Paul E. Parham, Edwin Michael

**Affiliations:** 1 Department of Infectious Disease Epidemiology, Imperial College London, London, United Kingdom; 2 MRC Centre for Outbreak Analysis and Modelling, Department of Infectious Disease Epidemiology, Imperial College London, London, United Kingdom; 3 Grantham Institute for Climate Change, Department of Infectious Disease Epidemiology, Imperial College London, London, United Kingdom; 4 Department of Biological Sciences, University of Notre Dame, Notre Dame, Indiana, United States of America; Emory University, United States of America

## Abstract

Malaria and lymphatic filariasis (LF) continue to cause a considerable public health burden globally and are co-endemic in many regions of sub-Saharan Africa. These infections are transmitted by the same mosquito species which raises important questions about optimal vector control strategies in co-endemic regions, as well as the effect of the presence of each infection on endemicity of the other; there is currently little consensus on the latter. The need for comprehensive modelling studies to address such questions is therefore significant, yet very few have been undertaken to date despite the recognised explanatory power of reliable dynamic mathematical models. Here, we develop a malaria-LF co-infection modelling framework that accounts for two key interactions between these infections, namely the increase in vector mortality as LF mosquito prevalence increases and the antagonistic Th1/Th2 immune response that occurs in co-infected hosts. We consider the crucial interplay between these interactions on the resulting endemic prevalence when introducing each infection in regions where the other is already endemic (e.g. due to regional environmental change), and the associated timescale for such changes, as well as effects on the basic reproduction number *R_0_* of each disease. We also highlight potential perverse effects of vector controls on human infection prevalence in co-endemic regions, noting that understanding such effects is critical in designing optimal integrated control programmes. Hence, as well as highlighting where better data are required to more reliably address such questions, we provide an important framework that will form the basis of future scenario analysis tools used to plan and inform policy decisions on intervention measures in different transmission settings.

## Author Summary

Malaria and lymphatic filariasis (LF) are thought to be co-endemic in many regions of Africa. Currently, most interventions targeted at these infections do not consider the impacts of co-infection. However, there have been increasing calls to adopt integrated control programmes that can achieve synergistic effects. Malaria and LF are both vector-borne diseases transmitted by *Anopheles* spp. mosquitoes, suggesting that well-designed vector control strategies have the potential to affect the transmission of both infections. In this study, we develop a modelling framework incorporating the specifics of malaria-LF co-infection to investigate how the transmission of each infection is altered for a range of possible interaction scenarios. We find that a control strategy that reduces LF transmission (via mass drug administration, for example) could potentially increase malaria prevalence. This work illustrates the potential perverse effects of targeting just one infection and emphasises the need to take into account co-endemic diseases when designing control programmes. The developed modelling framework can provide the basis for exploring the mix of options for joint control of these infections. We also highlight the need for better data on how co-infection impacts hosts and vectors in order for future predictions on both co-transmission dynamics and control to become more reliable.

## Introduction

Malaria and lymphatic filariasis (LF) cause the largest public health burden of all vector-borne diseases worldwide [1] with around 350–500 million clinical episodes and 1 million deaths every year caused by malaria [2] and more than 120 million people globally infected with LF. The diseases are co-endemic in many regions in sub-Saharan Africa and, importantly, are transmitted by the same vector species, the *Anopheles* spp. mosquito [3]. The infections can co-exist in both vectors [4], [5] and hosts [6]. Interactions between malaria and LF parasites are thought to have an effect on the transmission of both infections, in particular through changes in vector mortality as a result of either single infection or co-infection [7], [8]. Interactions in the host are likely to affect susceptibility and disease severity [9]–[13], and are determined by the effect the parasites have on immunological cytokines. Cytokines are proteins secreted by the immune system carrying signals to cells, mediating and regulating immunity, inflammation, and the development of blood cells. They are commonly divided into two categories – type 1 and type 2. Malaria is associated with a Th1 response, with increases in the production of type 1 cytokines, including IFN-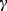 and TNF-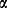 [14], which stimulate immunity and can result in extreme inflammatory responses. Lymphatic filariasis induces both Th1 and Th2 responses [15], [16]. Initially the response is Th1 biased, causing inflammation and protecting against incoming larvae. Subsequently, Th2 responses are induced, in particular cytokines IL-4, IL-10 and TGF-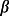 among others [14], [17], [18] which induce strong antibody responses and act to limit and contain infection. As infection progresses, Th2 levels increase, decreasing the Th1 response.

Understanding how co-infection affects the dynamics and control of important infectious diseases has become increasingly significant as evidence of meaningful within-host interactions between pathogens becomes better established [19]–[23]. Changes in the course of infection of co-infected individuals have been observed but, in general, the mechanisms of how transmission is altered are poorly understood. For example, HIV-infected individuals co-infected with Hepatitis C have been reported to experience a more rapid clinical progression compared to single infected individuals [24], [25]. Helminth infections are thought to exacerbate malaria symptoms by causing blood loss (and thus anaemia) and inhibiting the ability of the host to mount a Th1-type immune response [21]. These examples highlight two key ways in which co-infections can affect transmission and disease: each infection alters the ability of the immune system to adequately mount an immune response to the other infection, or one infection has a related symptom that exacerbates the associated symptoms and effects of the other infection.

Currently, there is little consensus on the effects of malaria and helminth co-infection on human hosts. There is evidence that co-infection can reduce [26], [27] or increase [14], [27], [28] malaria severity. A meta-analysis of 54 experiments conducted on laboratory mice investigating the effects of helminth infection on microparasite density suggested that the effect of interactions is dependent on the species pair [11], although none of the studies considered *Wuchereria bancrofti* (LF) and *Plasmodium* (malaria) co-infection. However, it is thought that infection with *W. bancrofti* increases mosquito susceptibility to *Plasmodium* infection, since migration of microfilariae disrupts the midgut, allowing *Plasmodium* easier access through the midgut to the salivary glands [3], [5], [29], [30]. On the other hand, mosquitoes carrying worm parasites have been found to reduce *Plasmodium* infectivity, with such vectors possessing a lower infection intensity compared to uninfected mosquitoes [31]. This suggests that reducing worm burden in a population could increase mosquito susceptibility to malaria infection. These mechanisms clearly need to be investigated further in natural worm-*Plasmodium*-*Anopheles* systems before inclusion in co-infection transmission models and we therefore do not consider this further here. Similarly, there is also evidence suggesting that *Plasmodium*-infected mosquitoes have higher numbers of *W. bancrofti* parasites [3], [29], [32]. A study in Papua New Guinea found that co-infected vectors are more common than we would expect from the prevalence of single infections [5], suggesting that infected vectors are more susceptible to other diseases. Co-infection has also been reported to affect the size, development and density of larvae and oocysts in the vector [32]. However, any advantages to disease transmission due to increased susceptibility may be lost by the reduction in survivorship caused by co-infection [5]. High levels of L3 larvae in co-infected vectors increases mortality, reducing the probability that vectors survive long enough to become infectious and transmit these diseases [3], [29], suggesting that this may be an important regulatory mechanism underlying co-transmission of malaria and filariasis.

The basic reproduction number, 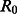, is a key concept in infectious disease epidemiology, which for a microparasite infection is defined as the average number of secondary cases generated per primary case in an entirely susceptible population. For a macroparasitic infection, 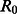 may be analogously defined as the average number of female offspring per adult female worm surviving to reproduction in the absence of density-dependence [33]. Consideration of this metric as a key measure of the transmission potential of either infection thus raises the question of how the reproductive potential of malaria or LF is modified in the presence of the other. In particular, a better understanding of this effect will be important for assessing the conditions under which either disease will successfully invade (and co-existence of both diseases may occur) into regions where the other is endemic. Given that we adopt a deterministic compartmental co-infection model here, the criteria for either disease to successfully invade reduces to the standard criterion 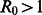 (while this represents a necessary, but not sufficient, condition in stochastic approaches).

Individually, malaria and LF models have been studied extensively [34]–[41], and these have frequently included analysis of key determinants of *R_0_*, but this study represents the first attempt to develop a combined LF and malaria transmission model. The modelling framework developed in this study is based on the hypothetical macroparasite-microparasite co-infection modelling framework developed in [42]. Explicitly modelling the interactions between malaria and LF is important for understanding how co-infection may impact the prevalence, reproduction number and elimination thresholds of both diseases, which clearly is also of import to quantifying the efficacy of integrated control approaches.

It has been suggested that targeting only LF may actually increase malaria incidence – LF infected mosquitoes have a higher mortality than uninfected mosquitoes due to the costs of larval burden [7], so eliminating LF increases vector lifespan, enabling greater malarial parasite transmission. Here, we develop a model of malaria and LF transmission to investigate (a) how these diseases, and their interactions, may be concurrently included in a consistent mathematical framework, (b) how effects due to parasites within hosts and vectors affect the baseline transmission dynamics of each disease in the presence of the other, and (c) how the basic reproduction number of each disease is affected by prevalence of the other.

## Materials and Methods

### Mathematical model background

A generic framework is developed in [42] for modelling microparasite-macroparasite co-infections, using a simple SI (susceptible-infected) model for microparasites in humans and a macroparasite model tracking the number of worms in hosts susceptible to, and infected with, the microparasite. Worms subsequently produce eggs that are released into the environment and may be passed to humans where they become adult macroparasites. We use the basic ideas behind this approach and apply these to the specific case of co-infection with malaria and LF. This involves several new additions including (1) explicitly modelling the vector population, with the external macroparasite infective stage represented as larvae in the vector, (2) dividing the human and vector populations into different compartments depending on malarial status and modelling the macroparasite population in each of these compartments, (3) including two parasite stages in the host, namely adult LF worms and microfilariae, (4) explicitly modelling the development of larvae in the vector, and (5) capturing the effect of co-infection on host infection dynamics.

### Microparasite model formulation

**Figure 1:**
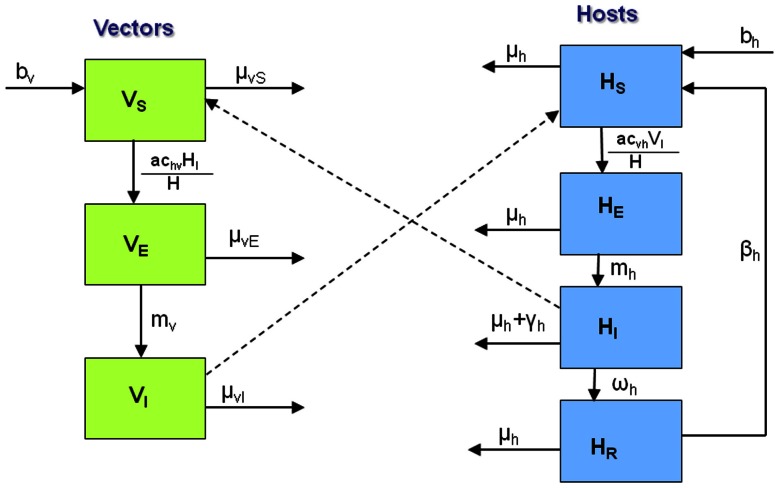
Basic structure of the malaria model. See Tables 1 and 2 for a summary of state variables and parameters.

The malaria component (Figure 1) of the full co-infection framework takes the form of an SEIRS model for human hosts (where we track the number of susceptible, exposed but not infectious, infectious, and recovered hosts, respectively denoted 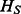, 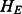, 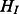 and 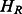) and an SEI model for vectors (with the number of vectors susceptible, exposed but not infectious, and infectious respectively denoted 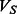, 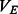 and 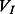), which are assumed not to recover from infection should it arise [34], [43]. Humans are assumed to be immune for a short duration after recovering from infection, before re-entering the susceptible population. Once an infectious mosquito bites a susceptible human and *Plasmodium* parasites enter the blood, the host is typically infected for several weeks before becoming infectious. If a susceptible vector bites an infectious human, it may become infected. The vector then becomes infectious at a rate dependent on the duration of the parasite sporogonic cycle (which is temperature-dependent, but typically takes around 12 days at 25°C [34]) and if it successfully bites a susceptible human, infection is passed on and the cycle continues.

The number of humans progressing from susceptible to exposed is determined by the force of infection from infectious vectors to susceptible hosts, and depends on the biting rate *a* (defined as the number of bites taken per vector per day) and the transmission probability of infectious vectors successfully transferring infection to susceptible humans. The rate at which humans move from exposed to infectious is determined by the duration of latency, and movement from infectious to recovered by the duration of infectiousness. Humans become susceptible again according to the rate at which immunity wanes. We also include host and vector births and deaths. Progression of vectors from susceptible to exposed is dependent on the force of infection from infectious hosts, while the progression from exposed to infectious is determined by the duration of the sporogonic cycle.

### Macroparasite model formulation

**Figure 2:**
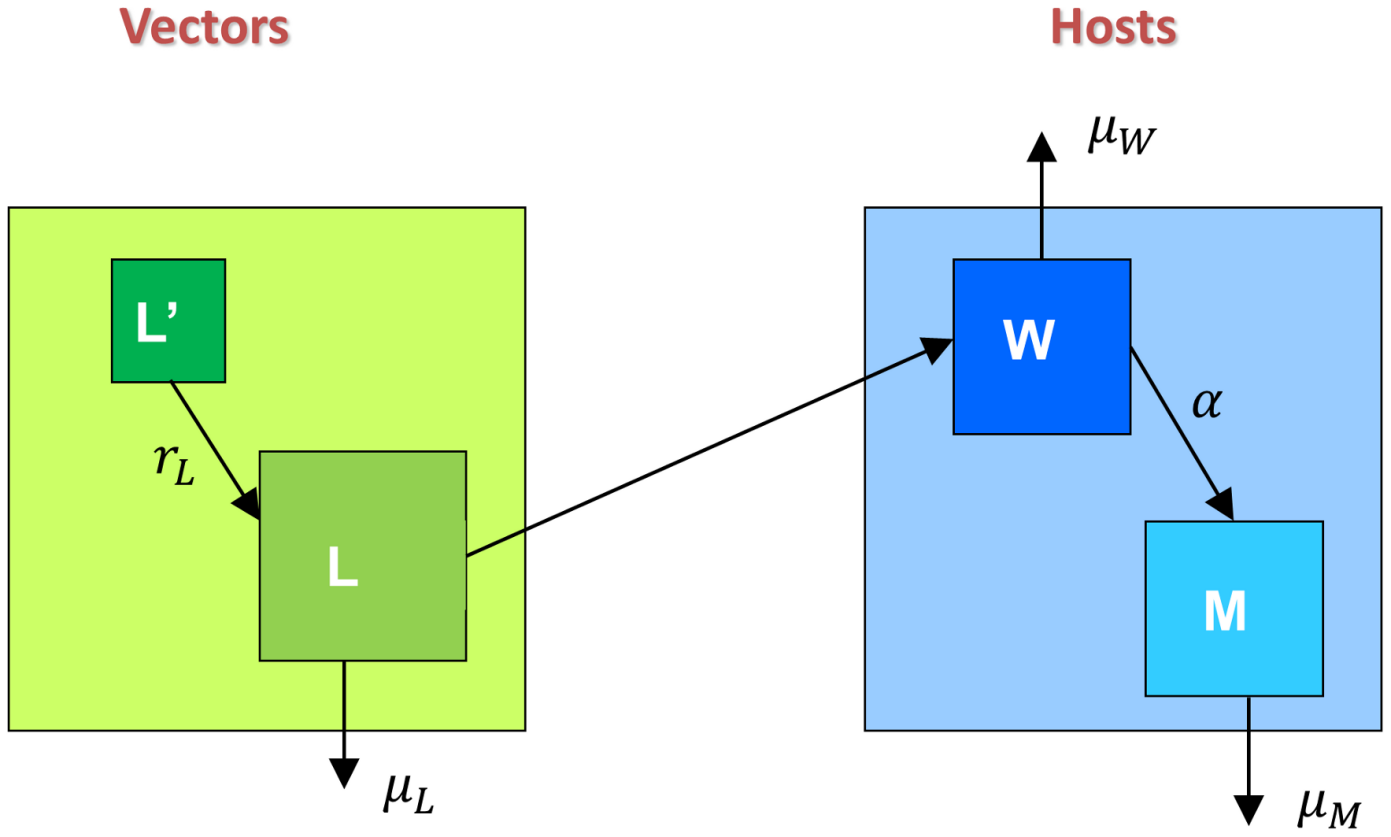
Basic structure of the LF model. See Tables 1 and 2 for a summary of state variables and parameters.

The LF component (Figure 2) of the full co-infection model is a simplified version of the model in [39] and extended in [40], where we assume age-independent LF transmission. We include an additional compartment in the model to represent the immature stages of larval development within vectors. Worms in the host produce microfilariae (mf), which may be ingested by biting mosquitoes and develop first into immature larvae, and then L3 larvae, before entering another human host at the next blood meal. These L3 larvae subsequently develop into worms in humans and the process continues. In this basic model, the number of worms, mf, immature larvae and L3 larvae are respectively denoted 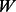, 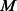, 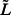 and 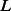.

### Full co-infection model formulation

**Figure 3:**
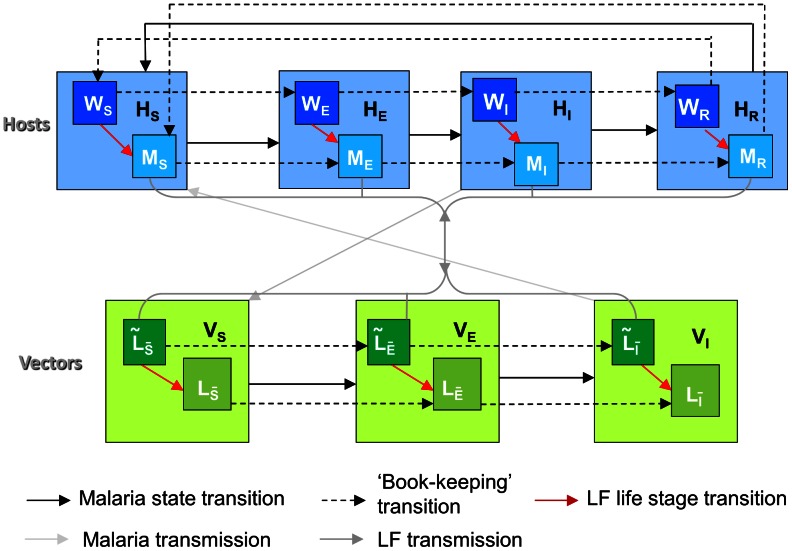
Structure of the full malaria-LF co-infection model. (NB. Life stage transition arrows from each *L* compartment to each *W* compartment should also strictly be present, but these are omitted here for clarity. All birth and deaths rates are also omitted, as well as the labelling of rates in terms of model parameters).

A simplified schematic (omitting all birth and deaths rates and the labelling of rates in terms of model parameters) of the full co-infection model is shown in Figure 3. The basic LF model is modified to account for the number of total worms and mf carried by hosts in each malaria compartment, and we thus track the total number of worms and mf in hosts who are susceptible, exposed (but not infectious), infectious and temporarily immune (recovered) from malaria. We denote these state variables 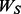, 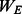, 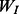 and 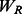 respectively for the worm burden and 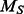, 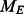, 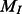 and 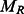 for the number of mf in humans. The number of new worms entering each worm compartment at each time step is determined by the biting rate of mosquitoes, the proportion of L3 larvae that leave mosquitoes and successfully enter the host, the total number of L3 larvae across all vectors, and the lower probability of worm development at higher total worm burden due to greater individual-level immunity. The total number of worms in each compartment is calculated by dividing the total worm burden up according to the proportion of hosts in each malaria compartment.We also account for the mortality of worms and humans, along with the movement of worms between compartments due to the changing malaria status of their hosts. The microfilaria equations are parameterised in terms of the number of mf produced per worm (per unit time per 20 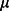
L of blood) and account for mf losses due to natural mf mortality, death of the host, and movement to new compartments due to changes in host malaria status.

Similarly, we track the number of underdeveloped larvae 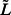 and fully-developed L3 larvae 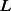 in vectors who are susceptible, exposed or infectious with respect to malaria. We denote these 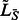, 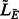 and 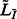 for immature larvae and 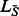, 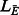 and 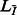 for L3 larvae respectively, where the overbar notation in the subscript emphasises reference to the malaria status of vectors, rather than humans. The number of larvae entering each developmental stage at each time step is determined by the biting rate, the probability that an mf enters a vector and successfully develops, and a density-dependent uptake function that governs the maximum number of mf that can be taken per mosquito. The rate of progression from underdeveloped larvae to developed L3 larvae is simply tracked by assuming a constant rate of progression. We include terms to account for the death of L3 larvae and immature larvae upon mosquito death, as well as larval movement between compartments as vectors change malaria status. Table 1 summarises state variables in the full model.

**Table 1:** State variables in the full co-infection model (where 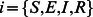 and 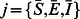).

State variable	Definition
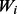	Number of worms in hosts with malaria status 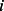 (where 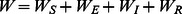 )
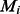	Number of microfilariae in hosts with malaria status 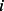 (where 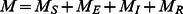 )
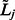	Number of larvae under development in vectors with malaria status 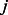 (where 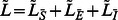 )
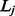	Number of larvae in vectors with malaria status 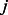 (where 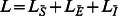 )
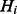	Number of hosts with malaria status 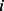 (where 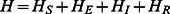 )
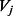	Number of vectors with malaria status 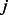 (where 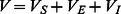 )

Interaction between the LF and malaria models occurs through (a) changes in mortality of mosquitoes infected with LF (assumed to be a linear hazard of L3 larval density), and (b) interaction between microparasites and macroparasites within hosts through the Th1/Th2 immune response, which affects the course of each infection (discussed further shortly).

Consider first the LF component of the model (see Figure 2). The number of worms and microfilariae in hosts are governed by the differential equations:
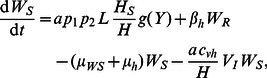
(1)

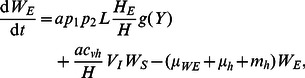
(2)

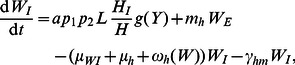
(3)

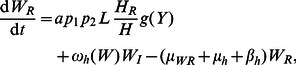
(4)

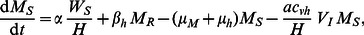
(5)

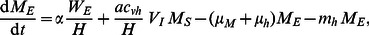
(6)

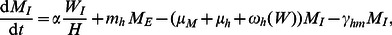
(7)

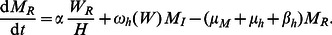
(8)In addition, the function 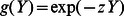 represents an immunity term for worms, where γ is a variable (which can be thought of as the ‘experience of infection’) that models the change in immunity over time as a function of worm burden per host and the current immunity level, and satsifies the equation 
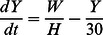
. LF prevalence in hosts can be calculated using 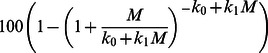 (where *k_0_* = 0.0029 and *k_1_* = 0.0236) [33]. For the number of immature and L3 larvae in vectors:
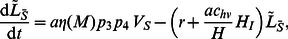
(9)

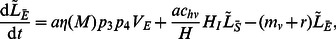
(10)

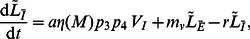
(11)

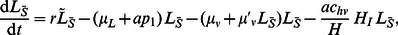
(12)

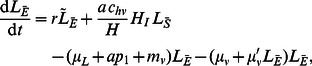
(13)

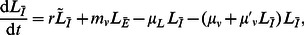
(14)where 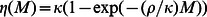 is the mf uptake function (with baseline values 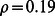 larvae per mf per 20 µL of human blood and 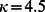 L3 larvae). For the malaria components of the model, the number of hosts and vectors in each compartment is given by:
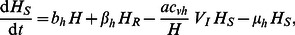
(15)

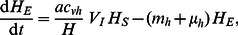
(16)

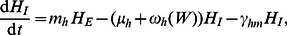
(17)

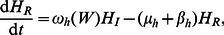
(18)

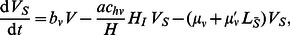
(19)

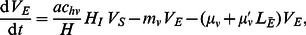
(20)

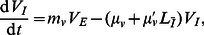
(21)where the effects of co-infection enter the malaria model through an increase in vector mortlaity due to the presence of larvae and the antagonistic Th1/Th2 response. Malaria prevalence in hosts is given by *H_I_*/*H*. A list of model parameters is given in Table 2.

**Table 2:** Parameters of the full co-infection model.

Parameter	Definition	Baseline value	Details
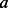	Biting rate (number of bites taken per vector per day)	0.2–0.5 days^−1^	Parameter varied to assume vectors bite on average once every 2 to 5 days
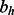	Birth rate of humans	1/18,250 days^−1^	Equal to host death rate to ensure constant population size
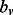	Birth rate of vectors	1/10 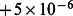 days^−1^	Equal to vector death rate to ensure constant population size
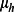	Death rate of humans	1/18,250days^−1^	Assumes host average life expectancy of 50 years
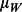	Death rate of worms	13/37500 days^−1^	[39]
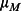	Death rate of microfilariae	1/300 days^−1^	[39]
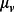	Death rate of vectors	0.1 days^−1^	Approximate mortality at 25°C [56]
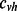	Probability of infectious vectors transferring infection to susceptible hosts	1/25	[57]
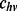	Probability of infectious hosts transferring infection to susceptible vectors	9/100	[57]
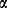	Production rate of mf per worm	1/15 days^−1^	[39]
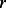	Rate of development from immature to L3 larvae	0.08 days^−1^	Development rate of [58] evaluated at 25°C
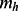	Rate at which infected hosts become infectious	1/10 days^−1^	[57]
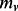	Rate at which infected vectors become infectious	0.02 days^−1^	Evaluation of 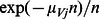 at *T* = 25°C (where 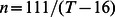 for *P. falciparum*) [34]
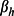	Rate at which humans return to susceptible from recovered	1/7 days^−1^	Host population assumed to experience loss of immunity within 1 week
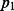	Proportion of L3 leaving mosquitoes per bite	0.414	[39]
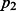	Proportion of L3 leaving mosquitoes that enter host	0.0003	[39], [59]
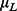	Death rate of larvae	0 days^−1^	Larval mortality assumed to arise only due to vector mortality
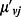	Additional mortality rate of larval-infected vectors with malaria status 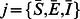	0.1 days^−1^	Uncertain parameter varied in sensitivity analysis (but within bounds to ensure realistic vector mortality at 25°C)
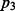	Probability of mf entering the vector upon biting an LF-infected host	0.37	[39]
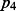	Probability of mf entering the vector developing into L3 larvae	1	[39] [25]
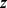	Force of immunity (strength of immune response)	0.2	[39], [59]

### Modelling LF/malaria interactions

#### Modelling LF/malaria interaction in the host

Th1 and Th2 responses are thought to be mutually inhibitory in the host and the interplay between these immune responses can determine the impact of co-infection. For example, LF infection may impair type-1 dependent control of malarial infection 14,44 because the anti-inflammatory cytokines IL-10 and IL-13, which are associated with helminth infection [27], inhibit the production of Th1 cytokines such as IFN-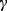 [44]–[46], suppressing the immune response against malaria [13] and resulting in a longer duration of infection. Th1 responses induced by malaria infection are thought to improve host ability to fight LF infection by increasing adult worm mortality. Here, we assume that Th1 and Th2 responses are antagonistic and that Th1/Th2 bias is determined by the mean worm burden in hosts – as worm burden increases, Th2 response is up-regulated and Th1 response down-regulated.

Immune responses are not directly modelled, but are instead captured by key parameters in the model. Th1 immune response to malaria is modelled by varying the recovery rate from malaria (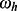) and Th2 response to LF modelled by varying worm mortality rate (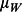) in hosts. Both parameters are assumed to be functions of worm burden. In co-infected hosts, when worm burden is low, immune response is Th1 skewed and the recovery rate from malaria (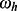) nears its maximum value, while 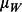 is high since the Th1 response increases worm mortality. As worm burden increases, immune response becomes more Th2-biased, Th1 levels decrease and worm mortality and malaria recovery rates decrease. We set realistic values for 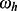 and 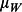 by defining minimum and maximum values for each parameter.

**Figure 4:**
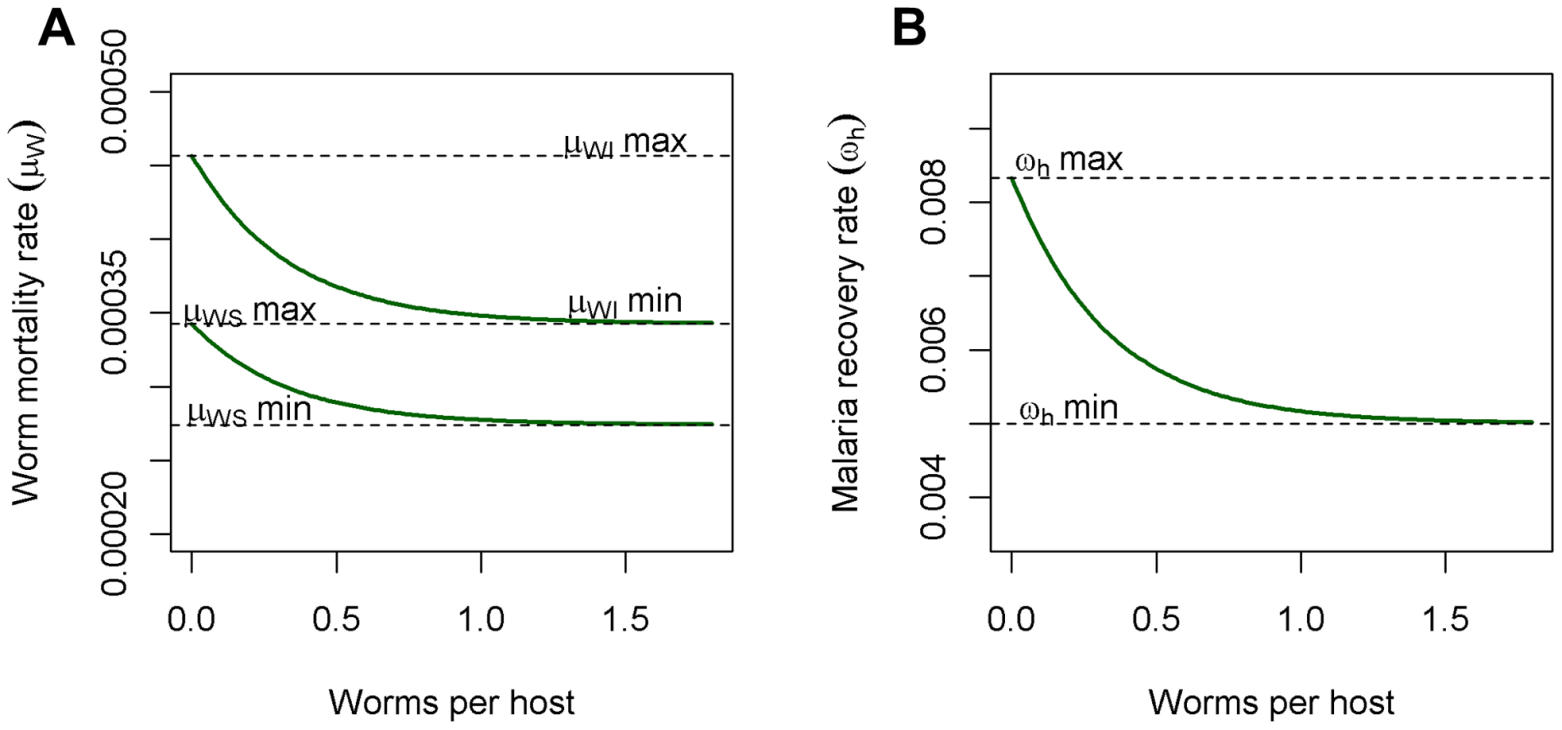
Worm mortality rate and malaria recovery rate as functions of mean worm burden. Increases in worm burden skew the immune system towards a Th2 response, lowering worm mortality rate (a) and human recovery rate (b) from malaria. The two curves in (a) represent the worm death rate in malaria-infected (upper) and malaria-susceptible human hosts (lower).

We assume that the immune response is more Th1 skewed in malaria infected individuals than malaria susceptible individuals and we represent this by using higher values for worm mortality in infected hosts, with the result that worms in infected hosts have a shorter mean lifespan. We parameterise these response as:
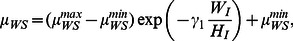
(22)

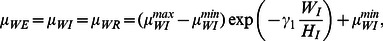
(23)

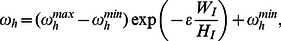
(24)where we assume baseline values 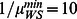 years, 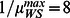 years, 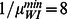 years, 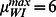 years, 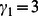 per worm, 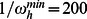 days, 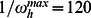 days and 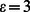 per worm. Figure 4 represents the effect of a reduction in Th1 response with increasing worm burden. It is unknown by how much increasing worm burden inhibits Th1 response, but it is thought that if parasites occupy distinct locations within the host, interactions may be smaller, meaning less Th1 inhibition [9]. The impact of increasing worm burden on worm mortality and malaria recovery can be altered by changing the slope of the response curves to explore different Th1/Th2 interaction scenarios, but for all simulations here, we use the functions shown in Figure 4.

#### Modelling LF/malaria interaction in the vector

Vectors are thought to experience larval-dependent mortality as a result of LF infection [47], [48]. Increased mortality with larval infection in *Culex* spp. mosquitoes has been reported in the literature – one study found that approximately 90% of uninfected vectors and 72% of infected vectors survive 16 days after taking a blood meal under laboratory conditions [48], while another found 21% higher mortality after feeding on mf-positive hosts compared to mf-negative hosts [49].

In *Anopheles*, however, there is little published data in this respect, while the limited available data, although indicating similar overall mortality rates to *Culex* (24% versus 27%), exhibits no association between mf intensity and mortality [8]. However, *Culex* have been studied more extensively and reports have shown increased mortality with larval density [50], [51]. Here, we assume a larval-dependent vector death rate, in addition to baseline mortality, so that the total mortality rate is 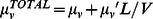, where 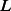 is the total number of larvae, 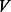 is the total number of mosquitoes, 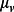 is the baseline death rate (i.e. with no larvae present), and 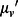 the magnitude of larval-induced mortality.

## Results

### Time-series behaviour of the full model

**Figure 5:**
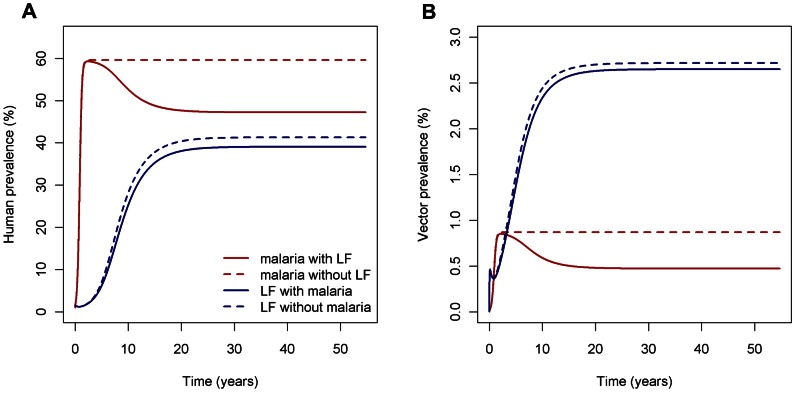
Malaria and LF prevalence in (a) humans and (b) mosquitoes with and without co-infection. Malaria and LF are introduced simultaneously into a population in the presence and absence of co-infection (with *a* = 0.2 day^−1^).

With the parameters in Table 2, Figure 5 contrasts the dynamics of malaria and LF with and without the presence of the other infection. We find that malaria prevalence is lower in humans when LF is present (Figure 5(a)). Host immunity is biased towards a Th1 response in the absence of LF, corresponding to a faster recovery from malaria and hence a decreased duration of infectiousness; however, while the presence of LF induces a greater Th2-skewed host immune response and would cause a slower *Plasmodium* clearance rate and hence increase in malaria prevalence if acting in isolation, this effect is less significant than larval-induced mortality decreasing vector life expectancy and hence the time for onwards malaria transmission. For the (realistic) parameter regime considered here, the net effect of LF presence is therefore to reduce malaria prevalence (although we note the importance of further experimental and field studies to address uncertainties in parameterisations of these two interactions to assess the generality and robustness of this result). The higher vector death rate when LF is present (Figure 5b) due to larval-induced mortality also results in a decrease in vector malaria prevalence and this again dominates over indirect effects due to changing host immune response.

Host LF prevalence is similarly reduced when malaria is present, since the presence of the microparasite elicits a more Th1-skewed response, resulting in higher filarial worm mortality in malaria-infected hosts and thus reduced LF prevalence. When malaria is absent, host immune response is Th2-biased and acts to sustain LF infection, rather than eliminate it, meaning that LF worms live for longer. In mosquitoes, LF prevalence is lower when malaria is present, since there are fewer worms in hosts and thus fewer mf per blood meal that may eventually develop into infective larvae.

**Figure 6:**
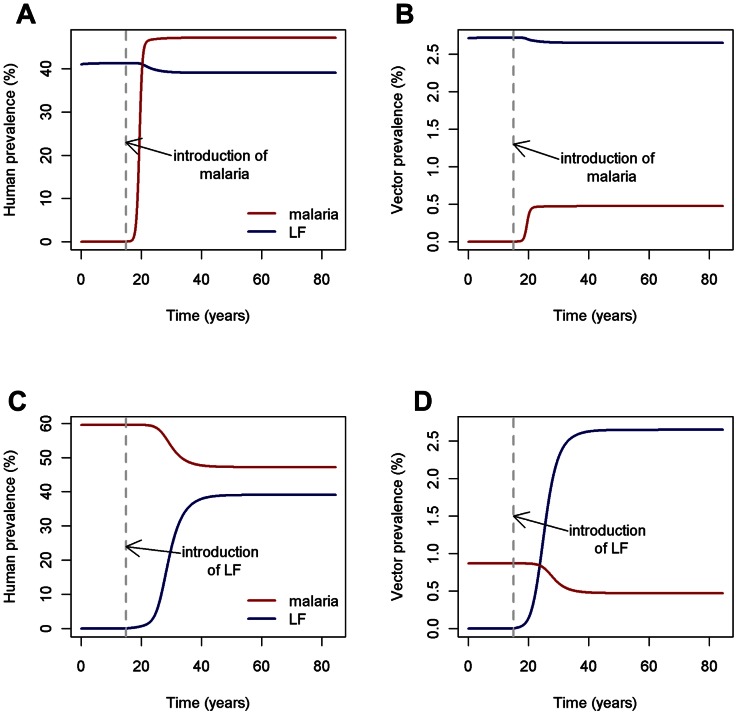
Invasion of (a and b) malaria in LF endemic regions, and (c and d) LF in malaria endemic regions. Prevalence time-series, in hosts and vectors, when introducing malaria or LF into endemic regions of the other.

The introduction of either infection into regions where the other is endemic alters the prevalence of the original infection. If malaria is introduced into an LF-endemic region, the prevalence of LF decreases marginally in both hosts and vectors (Figure 6), since worm mortality is higher in malaria-infected individuals, reducing overall worm burden. When LF is introduced into regions with endemic malaria, host and vector malaria prevalence decreases due to increased vector death rate resulting from LF larval-induced mortality, meaning that vectors have less time to complete sporogony and transmit malaria parasites.

We also note the speed at which both diseases reach a new equilibrium after introduction of the other infection. When malaria is introduced into LF-endemic regions, malaria prevalence quickly increases to its equilibrium level, while LF takes around 12 years to reach its new endemic state (Figure 6). Similarly, when LF is introduced into a malaria-endemic region, it subsequently takes around 30 years to reach its equilibrium, with malaria prevalence changing as soon as LF begins to increase significantly). These temporal differences are due to the significantly shorter lifecycle of malaria transmission compared to LF – the time taken for individuals to become infected with malaria and return to the susceptible class is less than a year, while LF worms can survive in hosts for around 10 years and mf can live for around 300 days. In addition, malaria transmission is more ‘efficient’ than LF transmission – only one bite is required from malaria-infected vectors to infect a human, whereas, on average, thousands of bites are needed from LF-infected vectors to produce one filarial worm. The dynamics of LF are therefore typically far slower and infections take longer to ‘take-off’. Furthermore, LF takes even longer to establish when malaria is present, since the immune response remains Th1-skewed at low LF prevalence, resulting in an increased worm mortality rate.

**Figure 7:**
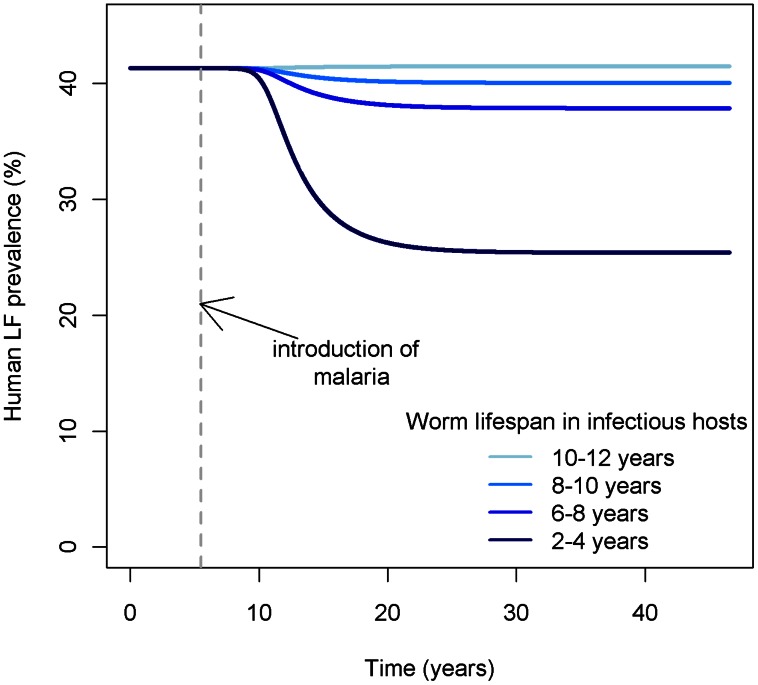
Human LF prevalence as a function of worm death rate in malaria-infected hosts. Considerable differences in LF prevalence are observed when malaria is introduced to the system depending on mean worm life expectancy.

These results are dependent on model parameters. We briefly explore the sensitivity of these results with respect to different assumptions regarding the worm lifespan in infectious hosts (Figure 7). Here, it is clear that when malaria is introduced into LF endemic regions, the magnitude of the reduction in LF prevalence depends on the extent to which malaria infection reduces worm lifespan in infectious hosts.

### The basic reproduction number 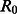


Various approaches may be used to derive 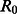, but arguably the most general is that of the next-generation approach introduced by [52], which holds, in principle, independent of model structure. Consider first the basic reproduction number of malaria in the presence of endemic LF, which we denote 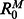. Following the formalism of [53], we consider equations (16), (17), (20) and (21) describing the dynamics of the infected compartments in the malaria component of the model, from which it is readily shown in Text S1 that calculating the dominant eigenvalue of the next-generation matrix gives
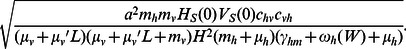
(25)Substituting for 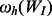, evaluating at the malaria-free equilibrium (where 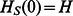, 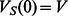 and 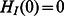), and defining 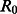 as the number of new infectious individuals produced by a single infectious individual in that class (which leads to the required 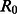 being the square of (25); see [53]) gives
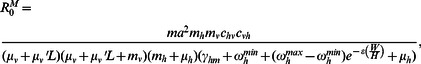
(26)which reduces to the standard 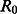 expression for malaria in the absence of LF (i.e. when 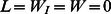) and where 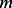 is the number of vectors per host. Thus, the reproductive potential of malaria is reduced by the presence of L3 larvae in infected and infectious mosquitoes (through decreased vector life expectancy), but increased due to the longer duration of host infectiousness (resulting from down-regulation of the Th1 response in the presence of LF) as mean worm burden in humans increases; further experimental data leading to more reliable estimation of *μ_v_'*, *ω_h_^max^*, *ω_h_^min^*, and *ε* will enable more robust quantitative conclusions to be drawn about the magnitude of these competing interactions on 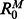 (Figure S1).

**Figure 8:**
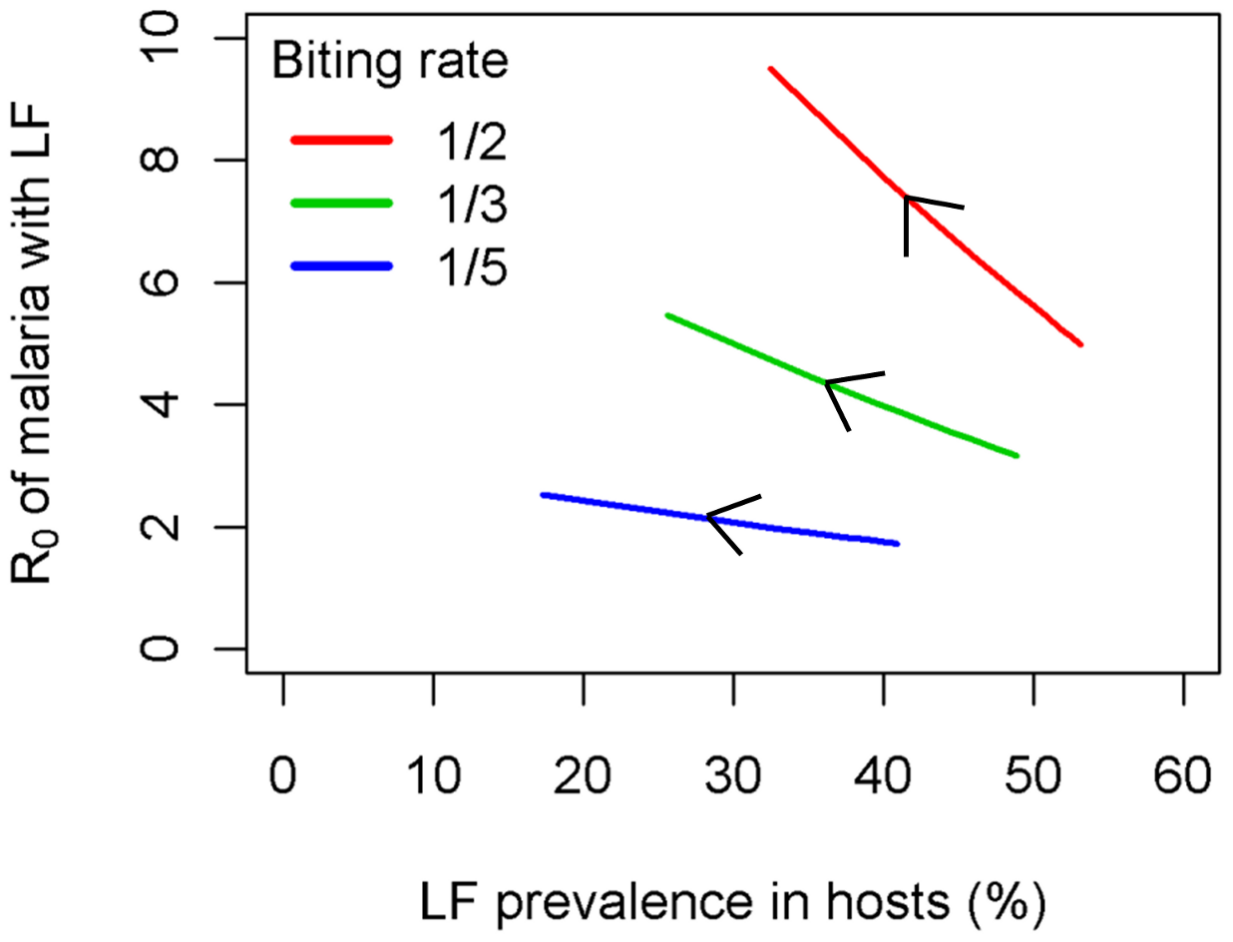
Dependence of *R_0_^M^* on LF prevalence in hosts for different mosquito biting rates.

To investigate how the *R*_0_ of malaria varies at different background levels of LF, we consider a range of biting rates (the largest of which are the most realistic) and vary the value of *μ_m_* (since this affects the equilibrium LF state, but without changing *R*_0_ *^M^*, as well as representing a parameter that may be influenced by LF controls) (Figure 8). The arrow on each biting rate curve denotes increasing *μ_m_* (where larger values imply shorter mf life expectancy and thus lower LF prevalence). Figure 8 confirms the analytical result from (13) that the *R*_0_ of malaria decreases as LF endemicity increases, and this effect becomes increasingly pronounced as the biting rate increases (with the steepest curves at the highest biting rates).

The drop in *R*_0_ *^M^* with increasing LF is consistent with Figure 5, which indicates that the introduction of LF causes malaria prevalence to decrease in humans and mosquitoes. In seeking a more complete understanding of the response of malaria transmission to LF presence across the full range of parameter space, however, it is nonetheless important to recognise that *R*_0_ *^M^* arises as the product of human and mosquito components of the parasite lifecycle. In some parameter regimes (not shown), introducing malaria into LF-endemic regions can increase the duration of human infectiousness (and hence human prevalence) due to a Th2-skewed host response, yet vector prevalence remains lower than if LF is not present due to the absence of larval-induced mortality. In this case, since the proportional drop in mosquito malaria prevalence is greater than the human component, the net effect is an overall drop in malaria prevalence (and hence *R*_0_), which is still consistent with (13), Figure 8, and standard theory on the monotonic relationship between *R*_0_ and endemic prevalence [33]. This subtle interplay between human and vector prevalence to produce the observed response of *R*_0_ *^M^* to LF presence is explored further in Figure S1 in Text S2 as a function of larval-induced vector mortality and human recovery from malaria.

An identical approach may be followed to calculate the basic reproduction number of LF in the presence of endemic malaria, which we denote 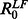, by considering equations (1)–(14) describing the LF component of the model. The need to track the number of worms and mf in four possible host states of malaria, together with the number of immature and L3 larvae within mosquitoes in three malaria states, results in a high-dimensional next-generation matrix (see Text S3). Evaluating this matrix at the LF-free equilibrium and calculating the dominant eigenvalue yields a closed-form solution for 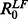 that is too unwieldy to reproduce here, but that we note reduces, in the absence of malaria, to
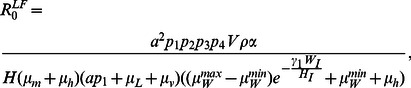
(27)a standard expression for the basic reproduction number of LF. In our model, the only effect of malaria presence on LF transmission is through the increased worm mortality rate in malaria infected hosts. However, since the reproduction number describes the number of new infections resulting from one primary infection in a totally susceptible population (and is thus a meaningful transmission metric for only a short duration before saturation effects occur), it is clear that the underlying malaria prevalence will not significantly affect 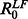 because new LF infections develop over a considerably shorter time span than the time taken for LF worms to die (even in malaria infected hosts where filarial worms experience a higher death rate relative to hosts susceptible to malaria). If malaria presence impacted microfilaria production or larval dynamics, we would expect 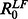 to be more strongly dependent on malaria prevalence, but in the absence of these effects, we obtain a very weak dependence (which does, nonetheless, marginally reduce LF prevalence in both humans and vectors and is thus consistent with Figure 5). Malaria does, however, alter LF transmission dynamics when both infections are endemic, as shown in Figures 5 and 6.

## Discussion

The model presented here, for malaria and LF co-infection within human and vector populations, represents the necessary groundwork towards a scenario analysis tool that could be used for policy planning. Three key interactions between the two parasites are introduced very simply into the basic interaction-free model through (1) increased mortality of vectors that are infected by either or both parasites, (2) increased mortality of LF worms in malaria co-infected hosts, and (3) increased recovery period from malaria in LF co-infected hosts (with the latter through modification of the human immune response towards one parasite in the presence of the other). These interactions, while reasoned judiciously here, are not expected to be comprehensive and are used here, along with plausible parameter values, to illustrate how these interaction terms (mortality rates and rates of recovery from infection) might appear in the expressions for *R*_0_ and subsequently impact the prevalence of each infection in the presence of the other. Nonetheless, there are other potentially important interactions between these infections. For example, as discussed earlier, there is the possibility of infection with one parasite affecting vectors' susceptibility to the other infection. Data on the effects of LF-malaria co-infections is almost non-existent; however, we can look at other helminth-malaria co-infections. In hosts, it has been reported that schistosomiasis can both increase and decrease the frequency of malaria attacks in co-infected individuals [54], [55], and that children with low intensities (but not higher intensities) of schistosomiasis have significantly lower *P. falciparum* densities than worm-free individuals [55]. Studies investigating how co-infection affects the course of each infection, as well as studies exploring the immune responses to co-infection, are needed to better inform the interactions assumed in our model. Overall, such scenario modeling is essential in an era of large scale Mass Drug Adminstration (MDA) and control programmes of tropical diseases, so that possible perverse effects are thought through in advance.

The results here show that perverse outcomes might be more complicated in a co-infection framework – even though the presence of one parasite appears to decrease the *R_0_* of the other in both cases, the *R_0_* calculated here is the overall value for the entire lifecycle and therefore takes into account human and vector components. The introduction of one parasite in the presence of the other may reduce the overall prevalence, but actually increase the prevalence in the humans while decreasing it in vectors in certain parameter regimes. Figure 6 shows that introducing LF reduces the prevalence of malaria, suggesting that, if LF was eliminated from a co-endemic region (using MDA, for example), this could actually result in an increase in malaria prevalence. These effects obviously need to be better understood so that inadvertant rises in human prevalence are avoided and improving our parameterisation of key interactions between malaria and LF epidemiology, such as those considered here, through new experimental studies is vital. In vectors, for example, better data on how susceptibility to infection and mortality are altered by co-infection are required. In humans, we need a better understanding of how the interplay between the Th1 and Th2 responses affect the ability of the host to mount an immune response to each infection, specifically, the impact of LF infection on the duration of malaria and whether or not malaria infection reduces the number of LF worms. We also need to find ways to translate the immune response findings from laboratory studies into meaningful assertions about how co-infections alter key epidemiological parameters in transmission models. In addition to these laboratory studies, parasite prevalence and intensity in communities with both infections should be monitored at high frequency prior to and during control programmes for high quality time-series to which epidemiological models such as ours can be fitted.

The factors influencing breakpoints – prevalence levels below which parasites become extinct – are the relative sizes of negative and positive density-dependent effects and the overall value of the reproduction number. In our model, the included density-dependencies are negative for the sake of simplicity, and breakpoints will not occur, but we plan to examine this important phenomenon in future work. Certainly, the changes induced in the LF reproduction number by the presence of malaria will alter the size of the breakpoint, though we have shown that the effect of malaria on the *R*_0_ of LF will be small for the interactions we include. An important next stage of our work is also to fit the parameters to data from field sites in which interventions have occurred and both infections have been monitored. Large increases in the malaria parasite rate in humans, following treatment for LF, would strongly determine the interaction parameters occuring in *R_0_^M^*, for example, as would the impact upon LF of the treatment for malaria. With commitment from international agencies and pharmaceutical companies to treat infectious tropical diseases, such data should become available soon and a parameterised modelling tool would then become invaluable.

## Supporting Information

Figure S1 **The contribution to overall malaria prevalence (and basic reproduction number) from host and vector populations as a function of larval-induced vector mortality and Th1/Th2 host immune response.** Panels A and B correspond to *μ_v_'* = 0.01 larvae vector^−1^ day^−1^, C and D consider *μ_v_'* = 0.1 larvae vector^−1^ day^−1^, and E and F represent *μ_v_'* = 1 larvae vector^−1^ day^−1^. Panels in the first column (ACE) correspond to *ω_h_* ^min^ = 1/180 day^−1^, while those in the second column (BDF) are run with *ω_h_* ^min^ = 1/360 day^−1^.
(TIF)

Text S1 **Derivation of the basic reproduction number of malaria in the presence of LF.**
(DOC)

Text S2 **Sensitivity analysis of contributions of infection levels in host and vector populations to overall malaria prevalence.**
(DOCX)

Text S3 **Derivation of the basic reproduction number of LF in the presence of malaria.**
(DOCX)
