# Supplementary figures and images for: Modelling Co-Infection with Malaria and Lymphatic Filariasis
Source: PLoS Comput Biol. 2013 Jun 13;9(6):e1003096. doi: 10.1371/journal.pcbi.1003096 (PMC3681634; doi:10.1371/journal.pcbi.1003096)

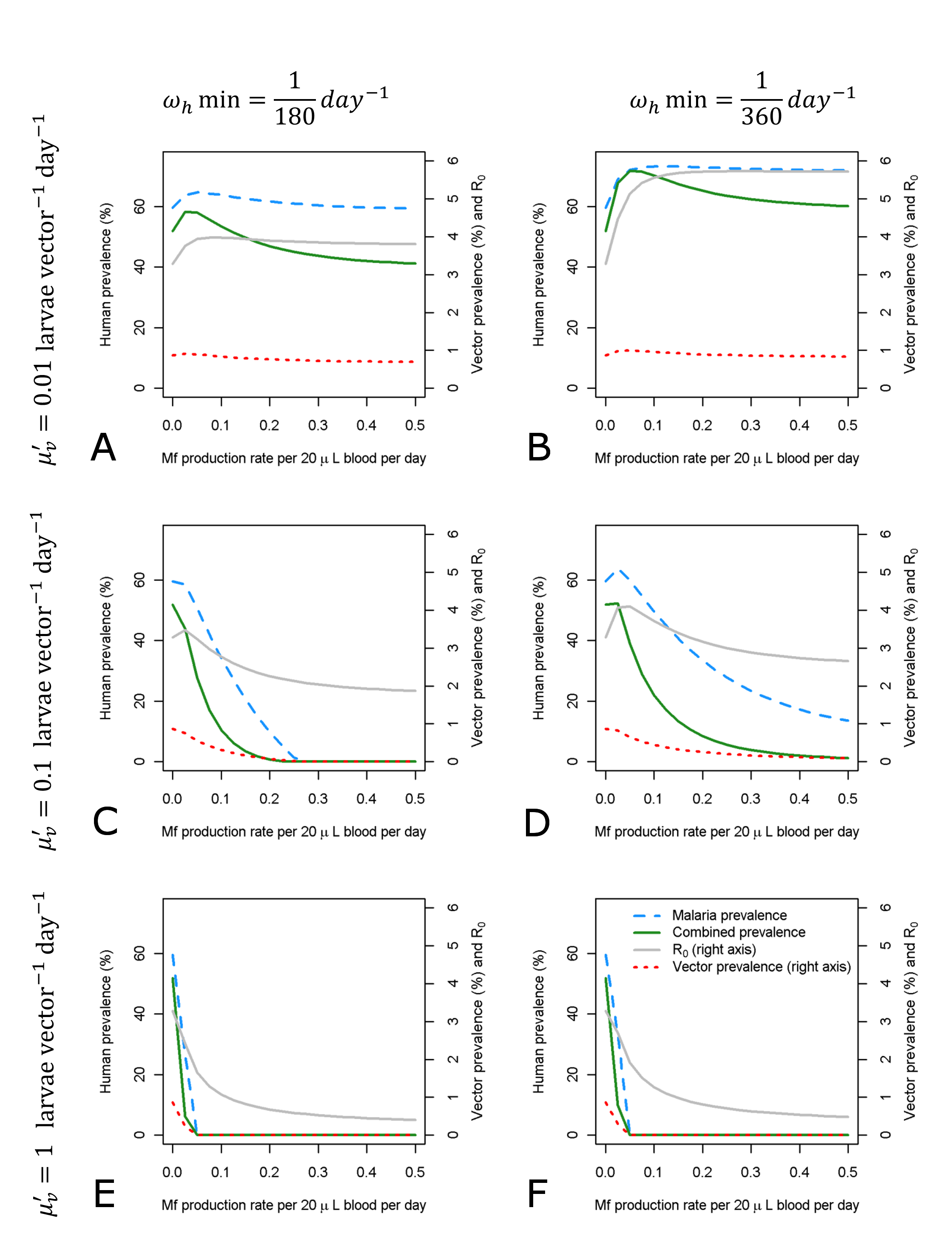

Supplement: Figure S1 — The contribution to overall malaria prevalence (and basic reproduction number) from host and vector populations as a function of larval-induced vector mortality and Th1/Th2 host immune response. Panels A and B correspond to μv' = 0.01 larvae vector−1 day−1, C and D consider μv' = 0.1 larvae vector−1 day−1, and E and F represent μv' = 1 larvae vector−1 day−1. Panels in the first column (ACE) correspond to ωh min = 1/180 day−1, while those in the second column (BDF) are run with ωh min = 1/360 day−1. (TIF) [file pcbi.1003096.s001.tif]
