# Supplementary material for: Modelling Co-Infection with Malaria and Lymphatic Filariasis
Source: PLoS Comput Biol. 2013 Jun 13;9(6):e1003096. doi: 10.1371/journal.pcbi.1003096 (PMC3681634; doi:10.1371/journal.pcbi.1003096)
Supplement: Text S1 — Derivation of the basic reproduction number of malaria in the presence of LF. (DOC) [file pcbi.1003096.s002.doc]

**Text S1 :** **Derivation of the Basic Reproduction Number of Malaria in the Presence of LF**

If we follow the formalism of Heffernan *et al.* (2005), consider the four equations decribing malaria infection in either humans or vectors, namely equations (16), (17), (20), and (21) in the main text. Tracking the number of new malaria infections through the matrix $\mathbf{F}$ (where $F_{1}=\frac{ac_{vh}V_{I}H_{S}}{H}$, $F_{2}=0$, $F_{3}=\frac{ac_{hv}H_{I}V_{S}}{H}$ and $F_{4}=0$) and all other flows of individuals into and out of compartments through the matrix $\mathbf{V}$ (so that $V_{1}=(m_{h}+\mu_{h})H_{E}$, $V_{2}=(\mu_{h}+\gamma_{hm}+\omega_{h})H_{I}-m_{h}H_{E}$, $V_{3}=(\mu_{v}+\mu_{v}'L+m_{v})V_{E}$ and $V_{4}=(\mu_{v}+\mu_{v}'L_{\bar{I}})V_{I}-m_{v}V_{E}$), we obtain

$$\mathbf{F}=\left(\begin{matrix} \frac{\partial F_{1}}{\partial H_{E}} & \frac{\partial F_{1}}{\partial H_{I}} & \frac{\partial F_{1}}{\partial V_{E}} & \frac{\partial F_{1}}{\partial V_{I}} \\ \frac{\partial F_{2}}{\partial H_{E}} & \frac{\partial F_{2}}{\partial H_{I}} & \frac{\partial F_{2}}{\partial V_{E}} & \frac{\partial F_{2}}{\partial V_{I}} \\ \frac{\partial F_{3}}{\partial H_{E}} & \frac{\partial F_{3}}{\partial H_{I}} & \frac{\partial F_{3}}{\partial V_{E}} & \frac{\partial F_{3}}{\partial V_{I}} \\ \frac{\partial F_{4}}{\partial H_{E}} & \frac{\partial F_{4}}{\partial H_{I}} & \frac{\partial F_{4}}{\partial V_{E}} & \frac{\partial F_{4}}{\partial V_{I}} \end{matrix}\right)=\left(\begin{matrix} 0 & 0 & 0 & \frac{ac_{vh}H_{S}(0)}{H} \\ 0 & 0 & 0 & 0 \\ 0 & \frac{ac_{hv}V_{S}(0)}{H} & 0 & 0 \\ 0 & 0 & 0 & 0 \end{matrix}\right)$$

and

$$\mathbf{V}=\left(\begin{matrix} \frac{\partial V_{1}}{\partial H_{E}} & \frac{\partial V_{1}}{\partial H_{I}} & \frac{\partial V_{1}}{\partial V_{E}} & \frac{\partial V_{1}}{\partial V_{I}} \\ \frac{\partial V_{2}}{\partial H_{E}} & \frac{\partial V_{2}}{\partial H_{I}} & \frac{\partial V_{2}}{\partial V_{E}} & \frac{\partial V_{2}}{\partial V_{I}} \\ \frac{\partial V_{3}}{\partial H_{E}} & \frac{\partial V_{3}}{\partial H_{I}} & \frac{\partial V_{3}}{\partial V_{E}} & \frac{\partial V_{3}}{\partial V_{I}} \\ \frac{\partial V_{4}}{\partial H_{E}} & \frac{\partial V_{4}}{\partial H_{I}} & \frac{\partial V_{4}}{\partial V_{E}} & \frac{\partial V_{4}}{\partial V_{I}} \end{matrix}\right)=\left(\begin{matrix} (m_{h}+\mu_{h}) & 0 & 0 & 0 \\ -m_{h} & (\mu_{h}+\gamma_{hm}+\gamma_{hf}W_{I}+\omega_{h}) & 0 & 0 \\ 0 & 0 & (\mu_{vE}+\mu_{vE}'L+m_{v}) & 0 \\ 0 & 0 & -m_{v} & \mu_{vI}+\mu_{vI}'L \end{matrix}\right),$$

which holds provided we make the approximation for $\omega_{h}(W_{I}/H_{I})$ in the main text. Forming the next-generation matrix $\mathbf{FV}^{-1}$ and calculating the dominant eigenvalue gives

$$\sqrt{\frac{a^{2}m_{h}m_{v}H_{S}(0)V_{S}(0)c_{hv}c_{vh}}{(\mu_{v}+\mu_{v}'L)(\mu_{v}+\mu_{v}'L+m_{v})H^{2}(m_{h}+\mu_{h})(\gamma_{hm}+\omega_{h}+\mu_{h})}},$$

which is (25) in the main text.
