# Supplementary material for: Modelling Co-Infection with Malaria and Lymphatic Filariasis
Source: PLoS Comput Biol. 2013 Jun 13;9(6):e1003096. doi: 10.1371/journal.pcbi.1003096 (PMC3681634; doi:10.1371/journal.pcbi.1003096)
Supplement: Text S2 — Sensitivity analysis of contributions of infection levels in host and vector populations to overall malaria prevalence. (DOCX) [file pcbi.1003096.s003.docx]

**Text S2 : Sensitivity Analysis of Contributions of Infection Levels in Host and Vector Populations to Overall Malaria Prevalence**

The graphs in Figure S1 demonstrate the competing effects of larval-induced vector mortality (which decreases the *R_0_* of malaria) versus the reduced recovery rate from malaria at high worm burdens due to the antagonistic Th1/Th2 effect. As $\mu_{v}^{'}$ increases from the top row to the bottom row (for fixed $\omega_{h}^{\min}$), vector mortality increases due to larval burden and this interaction plays a more significant role than modifications to host immunity caused by co-infection in driving malaria transmission dynamics and *R_0_^M^* (and human prevalence) is therefore reduced. As $\omega_{h}^{\min}$ decreases from the left column to the right column (corresponding to a stronger LF influence on the Th1 response to malaria in humans), the duration of infectiousness in hosts increases and thus *R_0_^M^* increases as worm burden becomes more significant. Overall, as well as illustrating the contribution of malaria prevalence in hosts and vectors to total prevalence, this set of figures highlight the sensitivity of conclusions about the effects of co-infection to model parameters quantifying the interactions considered in this paper, and hence the importance of obtaining better data on larval-induced vector mortality and host immune responses to more reliably assess how malaria and LF interact to produce observed patterns of transmission; we show here how only relatively small changes in $\mu_{v}^{'}$ and $\omega_{h}^{\min}$ may cause contrasting conclusions about how the presence of LF affects regional malaria prevalence. Other interactions (mentioned in the main text) may similarly compete with these two interactions at a host/vector level to affect population patterns of malaria transmission and we also highlight in the main text where such experimental and data-driven studies should perhaps be directed in order to further understand (and quantify) these effects.
