# Supplementary material for: Modelling Co-Infection with Malaria and Lymphatic Filariasis
Source: PLoS Comput Biol. 2013 Jun 13;9(6):e1003096. doi: 10.1371/journal.pcbi.1003096 (PMC3681634; doi:10.1371/journal.pcbi.1003096)
Supplement: Text S3 — Derivation of the basic reproduction number of LF in the presence of malaria. (DOCX) [file pcbi.1003096.s004.docx]

**Text S3:** **Derivation of the Basic Reproduction Number of LF in the Presence of Malaria**

Following an identical procedure to Supporting Information S2 by considering equations (1) – (14) in the main text, we first derive the matrix of new infections $\mathbf{F}$ where the elements $F_{ij}=\left(\frac{\partial F_{i}}{\partial X_{j}}\right)$ ($i,j=1,2,...14$) for

and the functions $F_{i}$ are given by $F_{1}=ap_{1}p_{2}L\left(\frac{H_{S}}{H}\right)g(Y)$, $F_{2}=a\eta(M)p_{3}p_{4}V_{S}$, $F_{3}=0$, $F_{4}=\frac{\alpha W_{S}}{H}$, $F_{5}=ap_{1}p_{2}L\left(\frac{H_{E}}{H}\right)g(Y)$, $F_{6}=a\eta(M)p_{3}p_{4}V_{E}$, $F_{7}=0$, $F_{8}=\frac{\alpha W_{E}}{H}$, $F_{9}=ap_{1}p_{2}L\left(\frac{H_{I}}{H}\right)g(Y)$, $F_{10}=a\eta(M)p_{3}p_{4}V_{I}$, $F_{11}=0$, $F_{12}=\frac{\alpha W_{I}}{H}$, $F_{13}=ap_{1}p_{2}L\left(\frac{H_{R}}{H}\right)g(Y)$, $F_{14}=\frac{\alpha W_{R}}{H}$ (with $\eta(M)=\kappa\left(1-e^{-\frac{\rho}{\kappa}M}\right)$). We can similarly calculate elements of the matrix $\mathbf{V}$ as $V_{ij}=\left(\frac{\partial V_{i}}{\partial X_{j}}\right)$ where $V_{1}=\left(\mu_{W}+\mu_{h}+\frac{ac_{vh}V_{I}}{H}\right)W_{S}-\beta_{h}W_{R}$, $V_{2}=\left(r+\frac{ac_{hv}H_{I}}{H}\right)\tilde{L}_{\bar{S}}$, $V_{3}=\left(\mu_{L}+\mu_{v}+\mu_{v}'L_{\bar{S}}+ap_{1}+\frac{ac_{hv}H_{I}}{H}\right)L_{\bar{S}}-r\tilde{L}_{\bar{S}}$, $V_{4}=\left(\mu_{M}+\mu_{h}+\frac{ac_{vh}V_{I}}{H}\right)M_{S}-\beta_{h}M_{R}$, $V_{5}=(\mu_{W}+\mu_{h}+m_{h})W_{E}+\frac{ac_{vh}V_{I}W_{S}}{H}$, , $V_{7}=(\mu_{L}+\mu_{v}+\mu_{v}'L_{\bar{E}}+ap_{1}+m_{v})L_{\bar{E}}-r\tilde{L}_{\bar{E}}-\frac{ac_{hv}H_{I}L_{\bar{S}}}{H}$, $V_{8}=(\mu_{M}+\mu_{h}+m_{h})M_{E}-\frac{ac_{vh}V_{I}M_{S}}{H}$, $V_{9}=(\mu_{W}+\mu_{h}+\omega_{h}(W)+\gamma_{hm})W_{I}-m_{h}W_{E}$, , $V_{11}=(\mu_{L}+\mu_{v}+\mu_{v}'L_{\bar{I}})L_{\bar{I}}-r\tilde{L}_{\bar{I}}-m_{v}L_{\bar{E}}$, $V_{12}=(\mu_{M}+\mu_{h}+\gamma_{hm}+\omega_{h}(W))M_{I}-m_{h}M_{E}$, $V_{13}=(\mu_{W}+\mu_{h}+\beta_{h})W_{R}-\omega_{h}(W)W_{I}$, $V_{14}=(\mu_{M}+\mu_{h}+\beta_{h})M_{R}-\omega_{h}(W)M_{I}$.

Substitituting $g(Y)=1$, evaluating the matrices at the LF-free equilibrium and forming the next-generation matrix $\mathbf{FV}^{-1}$ allows the dominant eigenvalue (and hence $R_{0}^{LF}$) to be calculated. Note also that the value of $R_{0}^{LF}$ that emerges from the next-generation approach needs to be raised to the power of three in order to be consistent with the standard definition of the basic reproduction number.
